# Mitotic CDK1 and 4E-BP1 II: A single phosphomimetic mutation in 4E-BP1 induces glucose intolerance in mice

**DOI:** 10.1371/journal.pone.0282914

**Published:** 2023-03-10

**Authors:** Simon Cao, Michael J. Jurczak, Yoko Shuda, Rui Sun, Masahiro Shuda, Yuan Chang, Patrick S. Moore

**Affiliations:** 1 Hillman Cancer Center, Cancer Virology Program, University of Pittsburgh, Pittsburgh, Pennsylvania, United States of America; 2 School of Medicine, University of Pittsburgh, Pittsburgh, Pennsylvania, United States of America; 3 Division of Endocrinology and Metabolism, University of Pittsburgh, Pittsburgh, Pennsylvania, United States of America; 4 Center for Metabolism and Mitochondrial Medicine, University of Pittsburgh, Pittsburgh, Pennsylvania, United States of America; Tohoku University, JAPAN

## Abstract

**Objective:**

Cyclin-dependent kinase 1 (CDK1)/cyclin B1 phosphorylates many of the same substrates as mTORC1 (a key regulator of glucose metabolism), including the eukaryotic initiation factor 4E-binding protein 1 (4E-BP1). Only mitotic CDK1 phosphorylates 4E-BP1 at residue S82 in mice (S83 in humans), in addition to the common 4E-BP1 phospho-acceptor sites phosphorylated by both CDK1 and mTORC1. We examined glucose metabolism in mice having a single aspartate phosphomimetic amino acid knock in substitution at the 4E-BP1 serine 82 (4E-BP1^S82D^) mimicking constitutive CDK1 phosphorylation.

**Methods:**

Knock-in homozygous 4E-BP1^S82D^ and 4E-BP1^S82A^ C57Bl/6N mice were assessed for glucose tolerance testing (GTT) and metabolic cage analysis on regular and on high-fat chow diets. Gastrocnemius tissues from 4E-BP1^S82D^ and WT mice were subject to Reverse Phase Protein Array analysis. Since the bone marrow is one of the few tissues typically having cycling cells that transit mitosis, reciprocal bone-marrow transplants were performed between male 4E-BP1^S82D^ and WT mice, followed by metabolic assessment, to determine the role of actively cycling cells on glucose homeostasis.

**Results:**

Homozygous knock-in 4E-BP1^S82D^ mice showed glucose intolerance that was markedly accentuated with a diabetogenic high-fat diet (p = 0.004). In contrast, homozygous mice with the unphosphorylatable alanine substitution (4E-BP1^S82A^) had normal glucose tolerance. Protein profiling of lean muscle tissues, largely arrested in G_0_, did not show protein expression or signaling changes that could account for these results. Reciprocal bone-marrow transplantation between 4E-BP1^S82D^ and wild-type littermates revealed a trend for wild-type mice with 4E-BP1^S82D^ marrow engraftment on high-fat diets to become hyperglycemic after glucose challenge.

**Conclusions:**

4E-BP1^S82D^ is a single amino acid substitution that induces glucose intolerance in mice. These findings indicate that glucose metabolism may be regulated by CDK1 4E-BP1 phosphorylation independent from mTOR and point towards an unexpected role for cycling cells that transit mitosis in diabetic glucose control.

## Introduction

Eukaryotic Initiation Factor 4E-Binding Protein 1 (4E-BP1), a kinase target for mechanistic target of rapamycin complex 1 (mTORC1) and cyclin-dependent kinase 1 (CDK1), regulates metabolic processes related to cellular energy levels and mitochondrial dynamics [1–4]. When 4E-BP1 is un-phosphorylated, it binds to the mRNA cap-binding protein eIF4E, preventing cap-dependent translation of specific mRNA targets [5]. Poly-phosphorylation of 4E-BP1 by mTORC1 during interphase dissociates 4E-BP1 from eIF4e, resuming cap-dependent protein synthesis [6].

During mitotic entry, CDK1 phosphorylates raptor, which shuts off mTORC1 activity [7]. CDK1 then subsumes the role of mTORC1 to phosphorylate some of the same targets as mTORC1, including 4E-BP1 [8–10]. Both mTORC1 and CDK1 phosphorylate 4E-BP1 at identical residues threonine (T)37, T46, T70, and serine (S)65, except only CDK1 additionally phosphorylates 4E-BP1 at S82 in mice (S83 in humans) [8, 11]. 4E-BP1 S82 phosphorylation by CDK1 is a robust molecular signature for mitosis [8, 9, 11] but its effect on 4E-BP1 function is unknown [11].

Both the mTOR pathway and 4E-BP1 play a critical role glucose tolerance and diabetes [12, 13]. Rapamycin inhibition of mTOR in rodents mimics caloric deprivation and is associated with decreased insulin sensitivity attributable to secondary effects on mTORC2 [12, 14, 15]. This effect has been referred to as “benevolent pseudo-diabetes” and may even be associated with increased longevity [16]. Concordantly, 4E-BP1 overexpression or activation has been linked to increased glucose tolerance in mice while knockout of 4E-BP1 and 4E-BP2 in mice results in insulin resistance, increased sensitivity to diet-induced obesity, and increased muscle lean mass and lipid accumulation [17–20]. The primary target of 4E-BP1, eIF4E, has also been shown to regulate lipid processing, storage, and weight gain in the context of a high fat diet in mice [21].

In this study, we find that a knock-in point mutation replacement of S82 with aspartic acid (S82D) in mice, but not replacement with alanine (S82A), results in reduced glucose tolerance in the setting of intact mTOR and CDK1 activity. These effects appear to be independent of perturbations to mTOR, CDK1, or insulin signaling pathways in lean muscle tissue as measured by reverse phase protein arrays (RPPA). Since the hematopoietic cell compartment is one of the few adult tissues with active cell cycling requiring CDK1/B1 activity, we performed bone marrow transplants between wild-type and transgenic knock-in S82D littermate mice. Glucose tolerance in transplanted mice was more similar to donor than to recipient genotypes in a high fat diet-induced insulin resistance model, suggesting an under-appreciated role for cycling hematopoietic cells in murine total body glucose regulation. Taken together, our findings lend support to studies showing that glucose intolerance in mice [12, 14, 17, 18] may be related to the phosphorylation status of 4E-BP1, particularly at 4E-BP1 residue S82, a residue that is phosphorylated by CDK1 but not mTOR.

## Materials and methods

### Experimental design

This study was designed to uncover metabolic defects in mice having a 4E-BP1 aspartate substitution (knock-in) point mutation at an amino acid site known to be phosphorylated by CDK1 during mitosis. Knock-in homozygous mice were compared to littermate control WT mice generated by heterozygous breeding. Defects in glucose tolerance for knock-in and control mice fed RCD and HFD were determined by glucose challenge, and metabolic changes were determined by housing in specialized metabolic cages. Longevity was determined by following a cohort of knock-in and control littermate mice under typical animal husbandry conditions until death or 20% peak weight loss.

### Generation of 4E-BP1^S82D^ and 4E-BP1^S82A^ knock-in mouse

Transgenic knock-in mice were generated by a commercial facility (Gen-O-Way) by homologous recombination. A targeting vector contained mutated exon 2 (4E-BP1^S82D^: S82_AGC_-to-D82_GAC;_ 4E-BP1^S82A^: S82_AGC_-to-A82_GCT_) with an upstream loxP-Neo-loxP cassette as well as both 5’ and 3’ intronic sequences surrounding the exon 2 (S1a Fig). Briefly, the targeting vector was introduced to the mouse embryonic stem (ES) cells and targeted cells were selected with G418. The successfully targeted ES cells were identified by southern hybridization (S1b and S1c Fig) and injected into blastocysts to develop the chimeric mice. The chimeric male mice were mated with C57BL/6 Cre female mice to excise the loxP-Neo cassette. Mice harboring germline-transmitted 4E-BP1^S82D^ or 4E-BP1^S82A^ allele were selected as the heterozygous founders, which are in a C57BL/6N genetic background. Finally, 4E-BP1^S82D^ and 4E-BP1^S82A^ homozygous mice were established by heterozygous breeding. The identity of 4E-BP1^S82D^ and 4E-BP1^S82A^ was verified by genotyping PCR (S1d Fig). Confirmation of the knock-in mutation and absence of off-target mutation was performed by whole genome sequencing.

### Mouse colony maintenance, irradiation and longevity studies

Mice breeding and long-term monitoring experiment was approved by the Institutional Animal Care and Use Committee (IACUC), University of Pittsburgh (IACUC experimental protocol#18012088). 4E-BP1^S82D^ homozygous and WT mice were established by 4E-BP1^S82D^/WT heterozygous breeding. The mice were fed a regular chow diet (RC; ProLab IsoPro RMH 3,000; kcal provided as approximately 26% protein, 14% fat, and 60% carbohydrate) though the longevity study. High-fat diet (HFD) was purchased from Research Diets (D12492) and provided kcal as approximately 20% protein, 60% fat and 20% carbohydrate. Body weight and changes in health condition were monitored weekly. Mice with 20% peak weight loss and/or severe illness were euthanized. For irradiation experiments, 11~13-week-old mice were subjected to total body irradiation at 9 Gy. Mice that survived from acute irradiation syndrome were monitored daily. Euthanasia criteria are the same as in long-term observation mice.

Per protocol, mice were sacrificed by using 100% CO_2_ followed by cervical dislocation if the mice developed tumors greater than 1.8 cm in diameter, or if the mice showed any signs of persistent mobidity such as loss in weight greater than 20%, lethargy, unwillingness to ambulate, hunched posturing and ruffled fur. No invasive procedures likely to produce moderate to severe pain were performed; 3–5% isoflurane inhalant induction and maintenance was performed for genotyping studies.

### Metabolic studies

The major determinants of whole-body energy balance were assessed in the Sable Systems Promethion Multi-plexed Metabolic cage system. Mice were individually housed in a home cage setting for 72 hours during which feeding, activity, energy expenditure and respiratory exchange ratio were continuously monitored. The first 24 h were considered acclimation and not included in the analysis such that data shown represent 48 h of data beginning on day two of housing. Body composition was measured by EchoMRI.

Glucose tolerance tests were performed after a 6 h morning fast (7am-1pm). Following collection of a basal blood sample (t = 0) by tail bleed, mice received an intraperitoneal bolus injection of glucose at 1.5 g/kg body weight followed by blood sampling at set intervals (15, 30, 45, 60 and 120 min) for blood glucose measurements and plasma insulin determination. Blood glucose was measured using a Bayer Contour Next glucometer and plasma insulin measured by chemiluminescence ELISA (Stellux, ALPCO).

### Bone marrow transplantation

Recipient mice were exposed to 10 Gy total body irradiation in a Shepherd Mark I Model 68 ^137^Cs gamma-irradiator (J.L. Shepherd & Associates) at a dose rate of ~70 Rad/min. Twenty hours after the irradiation of the recipient mice, donor mice were euthanized and whole bone marrow was flushed from femurs (18-gauge needle) and tibias (28-gauge needle) using cold PBS. Bone marrow was collected by centrifugation and red blood cells were lysed. Bone marrow cells were again collected by centrifugation, counted, and resuspended at 1 x10^7^ cells/mL (10 million/mL) in PBS. 1 x10^6^ (1 million) cells were injected into the tail vein of recipient mice. Bone marrow was fully reconstituted at 6–8 weeks and engraftment of the heterologous donor marrow was confirmed by bone marrow cell PCR [22].

### Protein isolation from tissue for reverse phase protein array (RPPA)

Tissue from the right gastrocnemius was isolated immediately following mouse sacrifice and frozen at -80°C until assayed. Frozen tissue was broken up by tweezers and collected in lysis buffer (1% Triton X-100, 150 mM NaCl, 1.5 mM MgCl_2_, 50mM HEPES, pH 7.4, 1 mM EGTA, 100 mM NaF, 10 mM Sodium Pyrophosphate, 1mM Na_3_VO_4_, 10% glycerol) [23] with freshly added protease inhibitor (cat. no. 05056489001; Roche) and phosphatase inhibitor (cat. no. 04906837001; Roche) within a Lysing Matrix D tube (cat. no. 116913050-CF; MP Biomedicals). Samples were homogenized using a FastPrep^®^ FP120 Cell Disrupter (Thermo Savant) at speed 6 in three 30 second intervals with incubation on ice for 5 minutes to prevent overheating. Supernatants were sonicated on ice 3 times for 5 seconds, and protein concentrations were quantified using Bio-Rad DC protein assay (cat. no. 5000112).

For RPPA proteomics, tissue lysate prepared in lysis buffer was diluted to a concentration of 1.5 μg/μl. Samples were mixed with 4x SDS sample buffer (40% glycerol, 8% SDS, 0.25M Tris-HCl, pH 6.8) with freshly added 10% β-mercaptoethanol [23]. Samples were boiled for 5 minutes and stored in -80°C. RPPA analysis and hierarchical clustering analysis was performed by the RPPA Core Facility at MD Anderson Cancer Center using methods described previously [23].

### Statistical analysis

PCA was performed in R using the stats (v3.6.2) package for the protein expression dataset generated by RPPA. The Kyoto Encyclopedia of Genes and Genomes (KEGG) database was used to obtain lists of proteins in the IIS (map04910), mTOR (map04150), and cell cycle (map04110) pathways [24]. Pathway-specific PCA utilized sets of RPPA dataset proteins and associated phosphorylations that corresponded to proteins in each pathway.

PRISM and R were used to produce graphs and perform statistical analyses using unpaired t-tests and log-rank tests, as appropriate. The R survminer v0.4.9 package was used to produce Kaplan-Meier plots. Adjusted P-values from RPPA data were calculated using Benjamini-Hochberg false discovery rate adjustment. P-value < 0.05 was considered significant.

## Results and discussion

### Generation and characterization of 4E-BP1^S82D^ and 4E-BP1^S82A^ knock-in mice

To examine the effects of a constitutively phosphorylated S82 site, we established C57Bl/6N homozygous mice with a presumed phospho-mimetic amino acid substitution of aspartate for serine (4E-BP1^S82D^) by homologous recombination (Genoway, France) (S1 Fig). Similarly, we produced mice expressing a constitutively unphosphorylated S82 site by substitution of alanine for serine (4E-BP1^S82A^). Aspartate substitution is commonly used to functionally mimic the negative charge of a phosphorylated amino acid [25], though it is unknown whether this is the case for 4E-BP1^S82D^. In 4E-BP1^S82A^ mice, amino acid 82 cannot be phosphorylated and remains uncharged regardless of cell cycle or CDK1 activity while mTOR or CDK1 phosphorylation at other 4E-BP1 sites remain unaffected. Correct knock-in transgenic mutation was confirmed by whole genome sequencing. No gross phenotypic changes were detected for the homozygous 4E-BP1^S82D^ and 4E-BP1^S82A^ knock-in mice, which had similar fertility and weight gain to wild-type (WT) littermates.

Per protocol, animals were sacrificed if reaching 20% peak weight loss, tumors greater than 2 cm^2^, disability, or distress. The overall survival of 4E-BP1^S82D^ mice was increased compared to WT littermates (29 mice per genotype, p = 0.03, log-rank test) (S2a–S2c Fig), but this was entirely attributed to WT male mice reaching end-point weight loss unexpectedly early rather than an increased longevity for 4E-BP1^S82D^ mice. WT male mice (n = 14) had a median endpoint survival that was less than WT female mice (n = 15, p = 0.01, log-rank test). In comparison, 4E-BP1^S82A^ mice overall survival was similar to that of 4E-BP1^S82D^ mice and was not significantly different from their littermate WT mice controls.

This experiment was initiated to determine the effects of human 4E-BP1 substitutions on susceptibility to neoplastic transformation, since expression of a constitutively dephosphorylated S83 site, 4E-BP1^S83A^, partially reverses rodent cell transformation induced by Merkel cell polyomavirus small T antigen viral oncoprotein [8, 9, 11]. No excess tumors occurred in S82D compared to wild-type littermates, but male 4E-BP1^S82D^ survival was diminished compared to WT males after 9 Gy irradiation (S3 Fig). No excess or unusual neoplasms were noted in either WT or 4E-BP1^S82D^ mice and further studies on tumorigenesis/survival after irradiation were not pursued. Gross pathology of internal organs, including liver and pancreas, was unremarkable for the 29 4E-BP1^S82D^ mice compared to 29 WT mice. Examination of liver sections by microscopy was similarly unremarkable and no tissue pathology was detected among S82D mice.

### Impaired glucose tolerance in male 4E-BP1^S82D^ but not 4E-BP1^S82A^ mice

To examine physiologic changes caused by the 4E-BP1^S82D^ knock-in mutation, eight 4E-BP1^S82D^ and eight WT littermate males aged 8–12 weeks were assessed by glucose tolerance testing (GTT) and metabolic cage analysis after conditioning for two weeks on a regular chow diet (RCD). One WT mouse had a short tail incompatible with GTT tail vein sampling and so was excluded from GTT but included in metabolic cage analyses. After GTT and metabolic cage studies, these mice were switched to a diabetogenic high fat diet (HFD) for 6 weeks pre-conditioning, and then GTT and metabolic analyses were repeated. Similarly, a separate cohort of eight 4E-BP1^S82A^ and nine WT littermate males aged 8–12 weeks were evaluated by GTT under RCD and HFD to compare the effects of a constitutively de-phosphorylated S82 site. Due to metabolic cage space constraints, one WT mouse was dropped at random (n = 8 in each group) for metabolic cage analysis of 4E-BP1^S82A^ and WT littermates.

**Fig 1:**
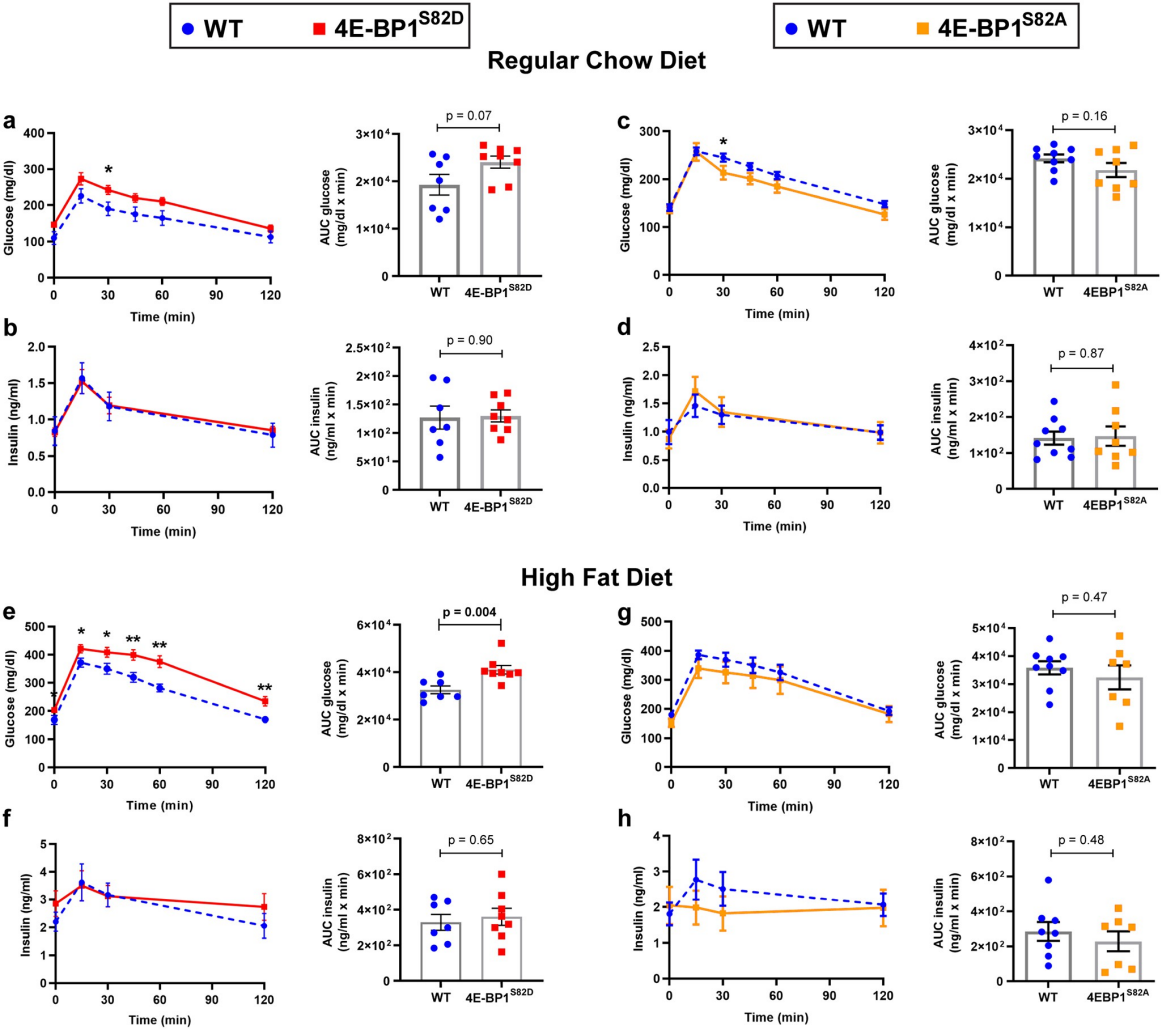
Glucose tolerance tests for male mice fed RCD and HFD showed reduced glucose tolerance for 4E-BP1^S82D^ mice but not 4E-BP1^S82A^ mice. 4E-BP1^S82D^ (n = 8) and WT (n = 7) male littermates were conditioned on RCD, and glucose tolerance tests (GTT) were performed with time course and area under the curve measurements for mouse serum glucose levels **(a)** and mouse serum insulin levels **(b)**. 4E-BP1^S82A^ (n = 8) and WT (n = 9) male littermates on RCD were similarly treated and measured for serum glucose **(c)** and insulin **(d)**. After HFD preconditioning for 6 weeks, glucose tolerance tests (GTT) were repeated on these mice with time course and area under the curve measurements for mouse serum glucose levels **(e, g)** and mouse serum insulin levels **(f, h)**. Mean and SEM are shown, and each plotted point represents one mouse. Two-tailed t-tests were used to compare groups. Comparisons at each data point were not significant (p≥0.05) unless noted with an asterisk. * p<0.05, ** p<0.01.

On RCD, GTT revealed a non-significant trend towards increased plasma glucose levels in the 4E-BP1^S82D^ group compared to WT male littermates throughout the 120 min study, with significantly elevated plasma glucose levels 30 min after glucose injection (Fig 1a). The area under the curve (AUC), however, was not significant (Fig 1a; p = 0.07, two-tailed t test). No differences in plasma insulin levels were detected (Fig 1b). In comparison, GTT for male 4E-BP1^S82A^ mice fed RCD showed no significant difference in plasma glucose or insulin levels compared to littermate WT mice (Fig 1c and 1d), with one time point of significantly lower plasma glucose levels in the 4E-BP1^S82A^ group at 30 min after glucose injection.

After conditioning on the HFD [26], fasting plasma glucose levels increased for both 4E-BP1^S82D^ and WT mice as expected (mean, respectively, 197 vs. 171 mg/dl, p = 0.27, two-tailed t test). GTT revealed significantly increased plasma glucose levels for 4E-BP1^S82D^ mice at each time point after glucose injection and significantly increased plasma glucose AUC, with no difference in serum insulin levels (Fig 1e and 1f). In contrast, HFD GTT for male 4E-BP1^S82A^ mice showed no significant difference in plasma glucose or insulin levels compared to littermate WT mice (Fig 1g and 1h). This is consistent with increased insulin resistance in 4E-BP1^S82D^ mice on HFD and may reflect an exacerbation of a modest phenotype seen on RCD. Notably, S82A mice having inactivating mutation at S82 showed reduced mean glucose levels at all time points than their wild-type controls on RCD and HFD GTT although this difference did not reach AUC significance.

In metabolic cage studies, RCD-fed 4E-BP1^S82D^ male mice had lower body weights compared to WT mice, with differences largely attributable to lower lean mass (S4a Fig), although ad libitum RCD-fed mice in the long-term survival study did not show significant body weight differences (S2d and S2e Fig). Under RCD, we detected no differences in energy expenditure or feeding when normalized to body weight, nor differences in respiratory quotient or total activity per mouse for 4E-BP1^S82D^ male mice (S4b–S4d and S5a–S5c Figs).

In metabolic cage studies of HFD-fed 4E-BP1^S82D^ mice, no significant differences in total body weight (S4e Fig) or normalized energy expenditure (S4f and S5d Figs) were present between 4E-BP1^S82D^ and WT mice. HFD-fed 4E-BP1^S82D^ mice displayed decreased overall feeding (due to decreases in feeding during the dark cycle) (S4g and S5e Figs) and a diminished respiratory quotient compared to WT mice during both light and dark cycles (S4h Fig), but showed no differences in activity (S5f Fig).

In contrast to the metabolic changes observed in 4E-BP1^S82D^ mice, 4E-BP1^S82A^ male mice fed RCD and HFD had no changes in the measured metabolic parameters (S4i–S4p and S5g–S5l Figs).

### Absence of significant protein changes between 4E-BP1^S82D^ and WT lean muscle tissues

On completion of HFD metabolic cage experiments, the S82D and WT littermate mice were sacrificed, and right gastrocnemius muscles were subjected to a 419 antibody Reverse Phase Protein Array (RPPA) analysis that includes most major known metabolic pathways having validated antibodies [23] to detect differential protein expression or phosphorylation. After controlling for false-discovery rate (FDR), no significant differences among any of the proteins/phosphoproteins was found (adj. p>0.05, S6a Fig).

On unadjusted analysis, 52 proteins showed differences between S82D and WT tissues (S1 Table), including raptor, which was reduced in 4E-BP1^S82D^ compared to WT muscle tissue (ratio: 0.94, p = 0.0024). These differences, however, were non-significant after FDR adjustment for multiple comparisons (p = 0.26). Gene ontology (GO) and pathway analysis based on unadjusted p-values < 0.05 using the DAVID bioinformatics resource [27] produced no significant GO term or pathway hits. Similarly, a Principal Components Analysis (PCA) and hierarchical clustering using all RPPA proteins revealed no distinct clustering for 4E-BP1^S82D^ or WT mice (S6b and S6f Fig). Insulin signaling/insulin-like growth factor, mTOR, and cell cycle/CDK1 pathway-specific PCA also displayed no apparent clustering (S6c–S6e Fig). Taken together, these RPPA data did not reveal lean muscle protein changes or known signaling effects consistent with changes to glucose metabolism for the 4E-BP1^S82D^ mice.

### Effect of bone marrow transplantation on the glucose intolerance phenotype

Since most cells in most non-embryonic tissues are arrested in G_0_, we sought to determine if reciprocal bone marrow transplantation, in which a portion of cells are actively cycling, could influence total plasma glucose response. Diabetes is a common sequela to human stem cell transplantation, but it is unknown if this is due to immunologic or transplantation drug effects [28, 29]. Conversely, data from mouse studies and case reports have demonstrated bone marrow transplantation as a method to potentially reverse type 1 and type 2 diabetic phenotypes, likely due to a stem-cell related mechanism [30–35].

Reciprocal bone-marrow transplants (BMT) were performed between 7–10 week old male 4E-BP1^S82D^ and WT mice. After a recovery period, surviving 20–23 week old male 4E-BP1^S82D^ mice with WT bone marrow (“4E-BP1^S82D^/marrowWT”) (n = 4) and WT mice with 4E-BP1^S82D^ bone marrow (“WT/marrow4E-BP1^S82D^”) (n = 4) mice were used for GTT. Spontaneous re-engraftment with 4E-BP1^S82D^ cells occurred in one 4E-BP1^S82D^ mouse transplanted with WT bone marrow and this mouse was dropped from the analysis. Expansion of this experiment was curtailed due to the emergence of the COVID-19 pandemic.

**Fig 2:**
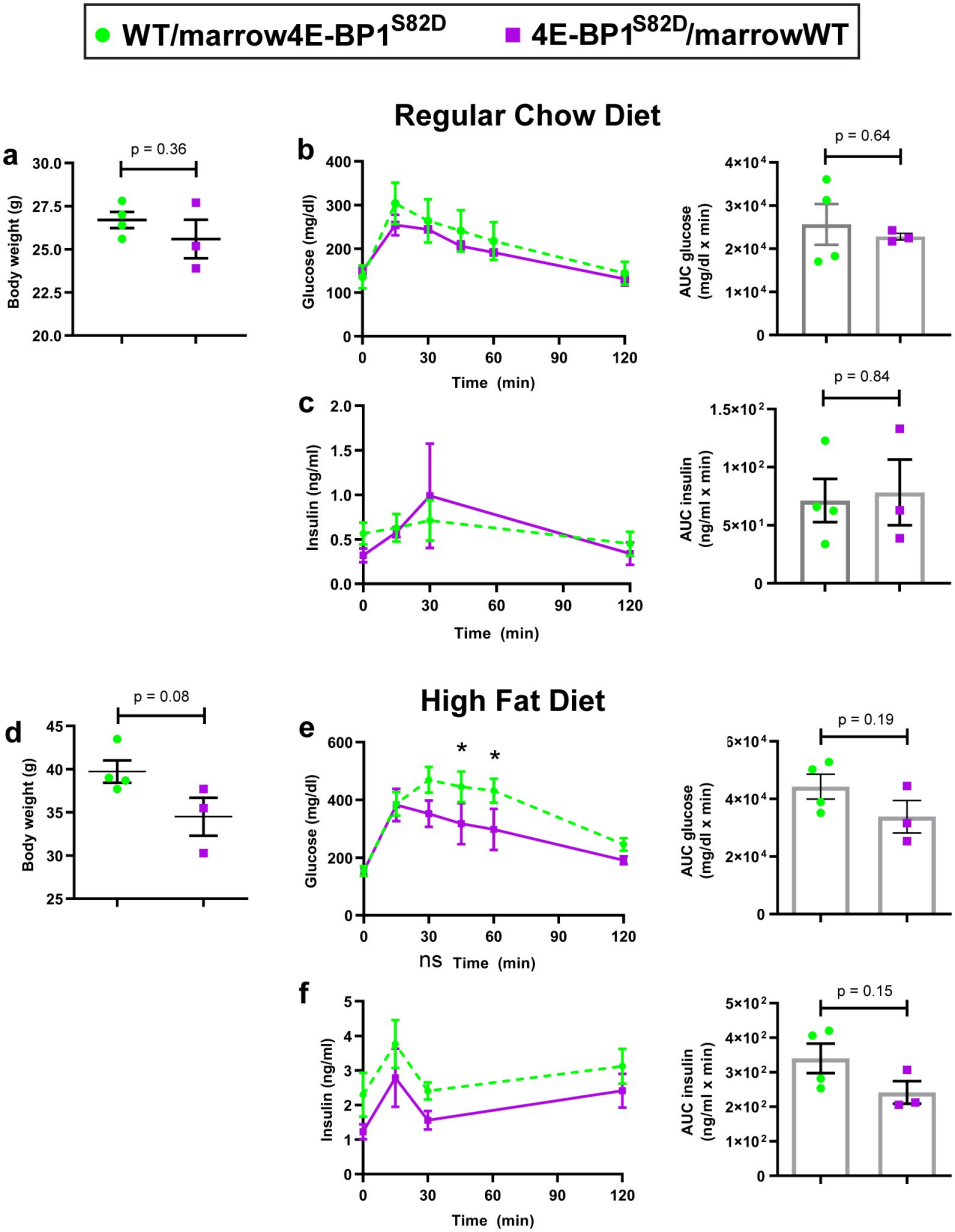
WT mice transplanted 4E-BP1^S82D^ bone marrow (WT/marrow4E-BP1^S82D^, n = 4) trend towards reduced glucose tolerance compared to 4E-BP1^S82D^ mice transplanted WT bone marrow (4E-BP1^S82D^/marrowWT, n = 3) after HFD challenge. After confirmation of successful reciprocal bone marrow transplantation, mice were preconditioned on RCD and measured for body weight prior to glucose tolerance test (GTT) **(a)**, serum glucose levels during GTT **(b)**, and serum insulin levels during GTT **(c)**. After switching from RCD to HFD, the mice were preconditioned on HFD and measured for body weight prior to glucose tolerance test (GTT) **(d)**, serum glucose levels during GTT **(e)**, and serum insulin levels during GTT **(f)**. Mean and SEM are shown, and each plotted point represents one mouse. GTT results shown as time course and area under the curve graphs. Two-tailed t-tests were used to compare groups. Comparisons at each data point were not significant (p≥0.05) unless noted with an asterisk. * p<0.05.

After RCD conditioning, no significant glucose tolerance differences were found for three 4E-BP1^S82D^/marrowWT mice and four WT/marrow4E-BP1^S82D^ mice (Fig 2b) and no difference in post-GTT insulin levels was observed (Fig 2c). After HFD-conditioning, WT/marrow4E-BP1^S82D^ mice showed an overall nonsignificant trend toward elevated glucose responses during GTT compared to 4E-BP1^S82D^/marrowWT (AUC glucose, p = 0.19 and AUC insulin level, p = 0.15, two-tailed t test) (Fig 2d and 2e), but WT mice receiving 4E-BP1^S82D^ marrow showed significantly elevated plasma glucose levels at 45 min (p = 0.0497) and 60 min (p = 0.039) after administration. In this small cohort, the comparative glucose intolerance trends seen with untransplanted 4E-BP1^S82D^ mice were attenuated (Fig 1).

Our data provide evidence that a single phosphomimetic amino acid substitution in 4E-BP1 promotes glucose intolerance on HFD. Our observed glucose intolerant phenotype is consistent with previously reported effects of 4E-BP1/2 knockout and the anticipated effects of 4E-BP1 inactivation by mTORC1-induced phosphorylation [12, 14, 17, 19, 20]. Phosphorylation of 4E-BP1 at S82, however, is regulated by CDK1, not mTOR, and the mTOR pathway was not significantly affected by 4E-BP1^S82D^ substitution as shown by RPPA analysis on mouse gastrocnemius muscle tissue. Expression of 4E-BP1 proteins for these two genotypes were indistinguishable, indicating that this effect is not due to loss of 4E-BP1 expression. Notably, circulating insulin levels did not significantly differ between S82D and WT mice prior to or during GTT.

Cell-based studies failed to resolve whether 4E-BP1^S82D^ or 4E-BP1^S82A^ substitutions alter cellular cap-dependent translation [11]. Cap-binding assays, for example, are unaffected in cells expressing these substituted proteins, which may indicate a cap-independent signaling role for CDK1 phosphorylation of 4E-BP1^S82^. Since this is only active during mitosis, 4E-BP1^S82^ phosphorylation is also likely to affect the small subset of cells (e.g., stem cells) actively undergoing cell division. In a separate study (under review PLoS One), 4E-BP1^S82A^ mice reveal profound polyscystic organ disease with aging that does not occur among 4E-BP1^S82D^ mice. Therefore, despite subtle effects of this mitotic CDK1 phosphorylation site mutation on cellular protein translation, this site has important physiological consequences to the whole animal.

Important caveats are needed for interpreting our data. Phospho-mimetics are imperfect molecular mimics for phosphorylation [25] so 4E-BP1^S82D^ substitution may not accurately model the biological effects of endogenous mitotic S82 phosphorylation. 4E-BP1^S82A^ substitution mutation did not lead to impaired glucose intolerance, providing confidence that a negatively-charged amino acid change at this site specifically contributes to a diabetic phenotype in mice. In contrast to CDK1 phosphorylation of S82, the aspartate substitution will introduce a negative charge at 4E-BP1 residue 82 that will persist throughout the cell cycle and in G0.

Second, our BMT studies on the role of the hematopoietic compartment in glucose tolerance involved small numbers of mice that did not allow clear statistical comparison. The results of these findings were consistent with the hematopoietic compartment having a disproportional impact on 4E-BP1^S82D^ genotype-related tolerance to a glucose challenge after a high-fat diet. We do not know if other cycling tissue compartments (e.g., skin, gut) also contribute to 4E-BP1^S82^-related glucose metabolism. Any conclusions from these experiments require confirmation due to the small sample numbers available in the BMT experiments.

Mitotic CDK1-mediated 4E-BP1 phosphorylation was found as a consequence of Merkel cell polyomavirus small T antigen sequestration of anaphase promoting complex [9, 36]. This results in 4E-BP1 multi-phosphorylation that is indistinguishable from interphase mTOR phosphorylation, except at residue S82 (S83 in humans). No differences in cap-dependent translation were found by eIF4G immunoprecipitation and refseq for human cell lines having S83 mutations [11]. Further, none of the single amino acid substitutions including S83D, S83E, and S83A in human 4E-BP1 affect 4E-BP1’s 7mGTP cap-binding activity in mitotic and asynchronous cell cycle conditions [8]. Taken together, these findings suggest two non-exclusive possibilities for a mechanistic target for S82D substitution: 1) the S82D substitution affects a tissue other than the gastrocnemius muscle sampled in our RPPA, or 2) S82D substitution affects a nontranslational signaling pathway that our RPPA panel did not interrogate.

The 4E-BP1^S82D^ mice serve as a precise genetic model to study the role of CDK1 and cell cycling in glucose metabolism. Whole gene knockout and drug therapies point towards the importance of 4E-BP1 phosphorylation to tumor suppression, longevity, and diabetic control [17, 18, 37–40]. These effects are often assumed to be related to mTOR activity. Here, we show that a point mutation at a site not targeted by mTOR reveals a novel role for 4E-BP1 in diabetes. While mTOR activity has been extensively examined in diabetic models, these data raise the possibility that CDK1 may also contribute to cellular regulation of glucose metabolism.

## Supporting information

S1 Fig Generation of 4EBP1^S82D^ knock-in mouse. **a)** Establishment of ES cells harboring heterozygous 4EBP1^S82D^ allele by homologous recombination. A targeting vector containing diphtheria toxin A (DTA), 5’ 4E-BP1 homology arm, loxP-Neo-loxP expression cassette, followed by S82D (AGC to GAC)-mutated 4E-BP1 exon (Ex) 2 and 3’ 4E-BP1 homology arm, was electroporated into ES cells to induce homologous recombination. Successfully targeted ES cells were injected into blastocyst and implanted into foster mother. Obtained chimeric male mice were mated with C57BL/6 Cre deleted female mice to excise loxP-Neo cassette, and littermate with 4EBP1^S82D^ germline transmission was selected as heterozygous founders. Restriction enzyme sites, size of expected fragments, and probes used for Southern hybridization are also indicated. **b)** Southern hybridization to confirm successful recombination in targeted ES cells after G418 selection. Genomic DNA digested with SpeI and AvrII was detected by probe A. The detection of a 5.1kb fragment indicates successful 5’ prime recombination. **c)** Southern hybridization using EcoN1-digested DNA detected by probe B. The detection of 7.2 kb fragment indicates successful recombination of 3’ homology arm. The targeted ES cells were injected into blastocyst and implanted into foster mother to generate highly chimeric male mice. **d)** Representative genotyping PCR results using genomic DNA extracted from ear notches. PCR primers flanking loxP site amplify 229 bp and 162 bp from 4EBP1^S82D^ mice and wild type (WT) littermate, respectively. Two fragments can be amplified from 4EBP1^S82D^/WT heterozygous littermate.
(TIF)

S2 Fig Male 4E-BP1^S82D^ mice lifespan is significantly longer than WT male mice lifespan with no significant difference in long-term body weight. Data were collected from long-term monitoring of mice on regular chow diet. Kaplan-Meier survival curves of **a)** Male and female 4E-BP1^S82D^ mice compared to WT mice (n = 29 per genotype). **b)** Male 4E-BP1^S82D^ mice (n = 16) compared to male WT mice (n = 14). **c**) Female 4E-BP1^S82D^ mice (n = 13) compared to female WT mice (n = 15). Average body weights for S82D and WT male **(d)** and female **(e)** mice did not significantly differ by genotype over time. Body weight was measured weekly from week 4 of life to mortality. Trendlines show weekly mean weight and 95% CI. Survival study statistical significance determined by log-rank test.
(TIF)

S3 Fig Male 4EBP1^S82D^ mice survival is significantly diminished after LD50 irradiation (9 Gy) compared to WT male mice. Kaplan-Meier survival curves of **a)** Male and female 4EBP1^S82D^ mice compared to WT mice (n = 18 per genotype). **b)** Male 4EBP1^S82D^ mice (n = 8) compared to male WT mice (n = 8). **c)** Female 4EBP1^S82D^ mice (n = 10) compared to female WT mice (n = 10). Statistical significance determined by log-rank test.
(TIF)

S4 Fig 4E-BP1^S82D^ mutation leads to metabolic differences among mice fed RCD and HFD, but 4E-BP1^S82A^ mutation does not. In metabolic cage experiments, male 4E-BP1^S82D^ and WT littermates (n = 8 for each genotype) were fed RCD and were measured for body weight (BW), lean mass, and fat mass **(a)**, energy expenditure per kg BW **(b)**, feeding per kg BW **(c)**, and respiratory quotient per mouse **(d)**. Then, mice previously fed RCD were pre-conditioned with HFD for 6 weeks prior to metabolic analyses and were measured for body weight, lean mass, and fat mass **(e)**, energy expenditure per kg BW **(f)**, feeding per kg BW **(g)**, and respiratory quotient per mouse **(h)**. Metabolic cage experiments were repeated in 4E-BP1^S82A^ and WT littermates fed RCD **(i-l)** and then HFD **(m-p)** (n = 8 for each genotype). Mean and SEM are shown, and each plotted point represents one mouse. Two-tailed t-tests were used to compare groups.
(TIFF)

S5 Fig Per mouse energy expenditure, feeding, and total activity metabolic cage data. Additional per-mouse metabolic cage data for energy expenditure, feeding, and total activity are shown here to accompany data presented in S4 Fig. Male 4E-BP1^S82D^ and WT littermates (n = 8 for each genotype) were fed RCD and were measured for energy expenditure per mouse **(a)**, feeding per mouse **(b)**, and total activity per mouse **(c).** After reconditioning on HFD, the mice were measured for energy expenditure per mouse **(d)**, feeding per mouse **(e)**, and total activity per mouse **(f).** Metabolic cage experiments were repeated in 4E-BP1^S82A^ and WT littermates fed RCD **(g-i)** and then HFD **(j-l)** (n = 8 for each genotype). Mean and SEM are shown, and each plotted point represents one mouse. Two-tailed t-tests were used to compare groups.
(TIF)

S6 Fig Gastrocnemius muscle from 4EBP1^S82D^ mice do not display clear protein-level differences from WT mice. Right gastrocnemius muscle tissue from male mice subject to GTT (4EBP1^S82D^ n = 8, WT n = 7) were used for reverse-phase protein array (RPPA). **a)** Volcano plot of protein expression by -log(FDR adj. p-value) and expression ratio. **b-e)** Principal component analysis (PCA) of protein expression in 4EBP1^S82D^ and WT mice from proteins in the full RPPA dataset **(b)**, insulin signaling pathway (IIS, **c**), mTOR signaling pathway **(d)**, or cell cycle pathways **(e)**. **f)** Hierarchical clustering of 4EBP1^S82D^ and WT mice based on protein expression from the full RPPA dataset.
(TIF)

S1 Table RPPA using 419 antibodies on right gastrocnemius muscle tissue from 4EBP1^S82D^ (n = 8) and WT (n = 7) mice show 52 proteins with unadjusted p-value < 0.05 for difference in expression. No false discovery rate (FDR)-adjusted p-values were significant.
(XLSX)

S1 File Reverse phase protein array results datafile. (XLSX)

S1 Raw images (TIFF)
