# Supplementary figures and images for: Mitotic CDK1 and 4E-BP1 II: A single phosphomimetic mutation in 4E-BP1 induces glucose intolerance in mice
Source: PLoS One. 2023 Mar 10;18(3):e0282914. doi: 10.1371/journal.pone.0282914 (PMC10004604; doi:10.1371/journal.pone.0282914)

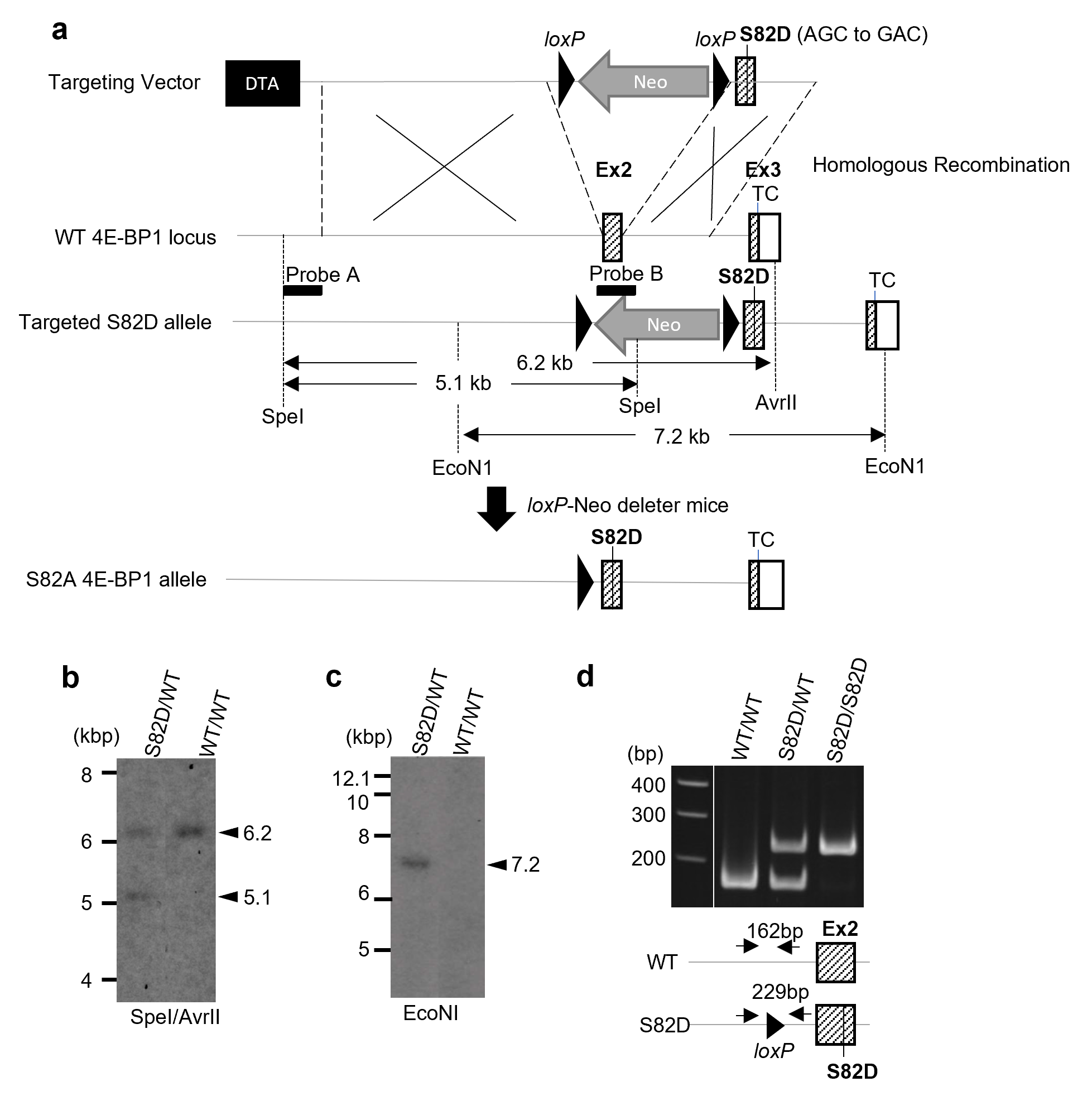

Supplement: S1 Fig — a) Establishment of ES cells harboring heterozygous 4EBP1S82D allele by homologous recombination. A targeting vector containing diphtheria toxin A (DTA), 5’ 4E-BP1 homology arm, loxP-Neo-loxP expression cassette, followed by S82D (AGC to GAC)-mutated 4E-BP1 exon (Ex) 2 and 3’ 4E-BP1 homology arm, was electroporated into ES cells to induce homologous recombination. Successfully targeted ES cells were injected into blastocyst and implanted into foster mother. Obtained chimeric male mice were mated with C57BL/6 Cre deleted female mice to excise loxP-Neo cassette, and littermate with 4EBP1S82D germline transmission was selected as heterozygous founders. Restriction enzyme sites, size of expected fragments, and probes used for Southern hybridization are also indicated. b) Southern hybridization to confirm successful recombination in targeted ES cells after G418 selection. Genomic DNA digested with SpeI and AvrII was detected by probe A. The detection of a 5.1kb fragment indicates successful 5’ prime recombination. c) Southern hybridization using EcoN1-digested DNA detected by probe B. The detection of 7.2 kb fragment indicates successful recombination of 3’ homology arm. The targeted ES cells were injected into blastocyst and implanted into foster mother to generate highly chimeric male mice. d) Representative genotyping PCR results using genomic DNA extracted from ear notches. PCR primers flanking loxP site amplify 229 bp and 162 bp from 4EBP1S82D mice and wild type (WT) littermate, respectively. Two fragments can be amplified from 4EBP1S82D/WT heterozygous littermate. (TIF) [file pone.0282914.s001.tif]

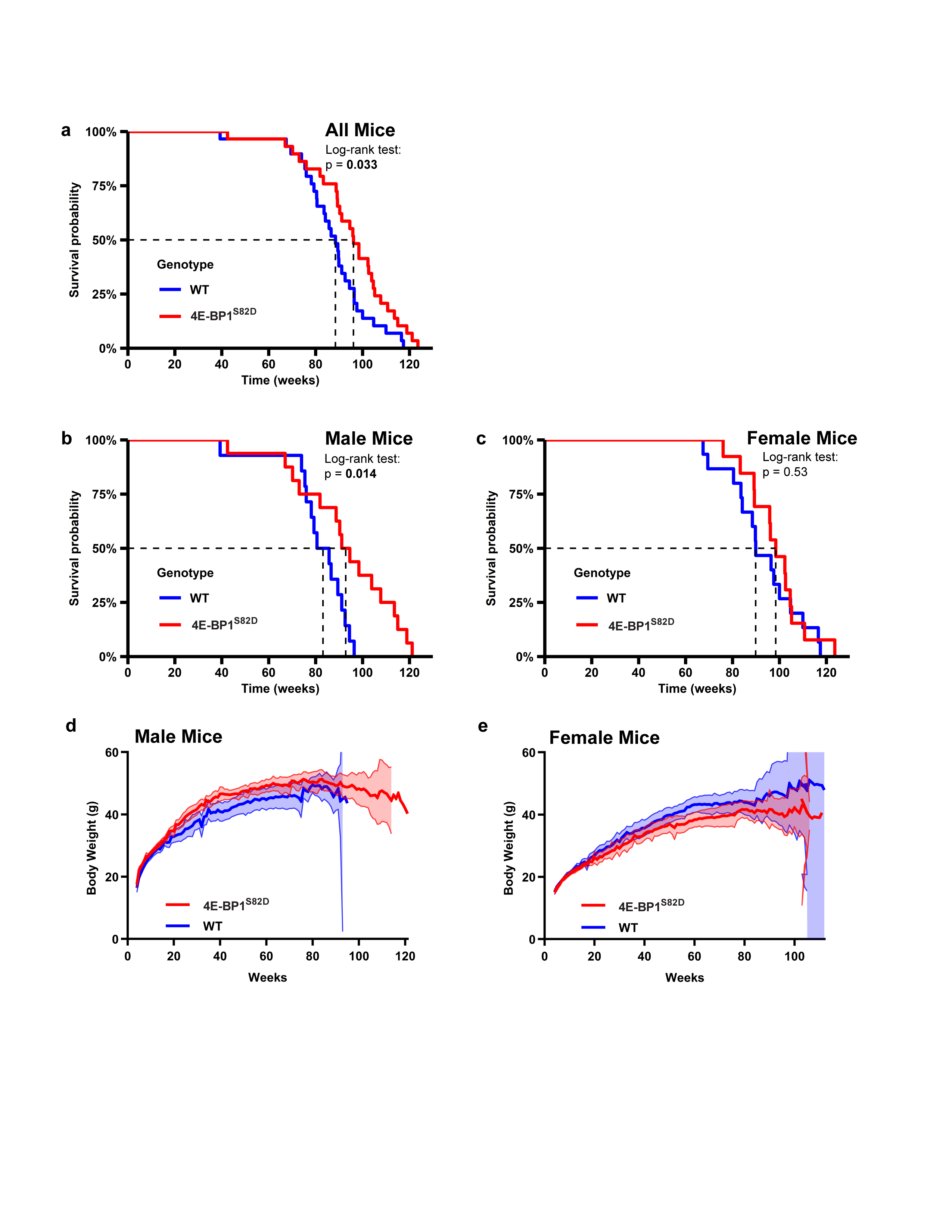

Supplement: S2 Fig — Data were collected from long-term monitoring of mice on regular chow diet. Kaplan-Meier survival curves of a) Male and female 4E-BP1S82D mice compared to WT mice (n = 29 per genotype). b) Male 4E-BP1S82D mice (n = 16) compared to male WT mice (n = 14). c) Female 4E-BP1S82D mice (n = 13) compared to female WT mice (n = 15). Average body weights for S82D and WT male (d) and female (e) mice did not significantly differ by genotype over time. Body weight was measured weekly from week 4 of life to mortality. Trendlines show weekly mean weight and 95% CI. Survival study statistical significance determined by log-rank test. (TIF) [file pone.0282914.s002.tif]

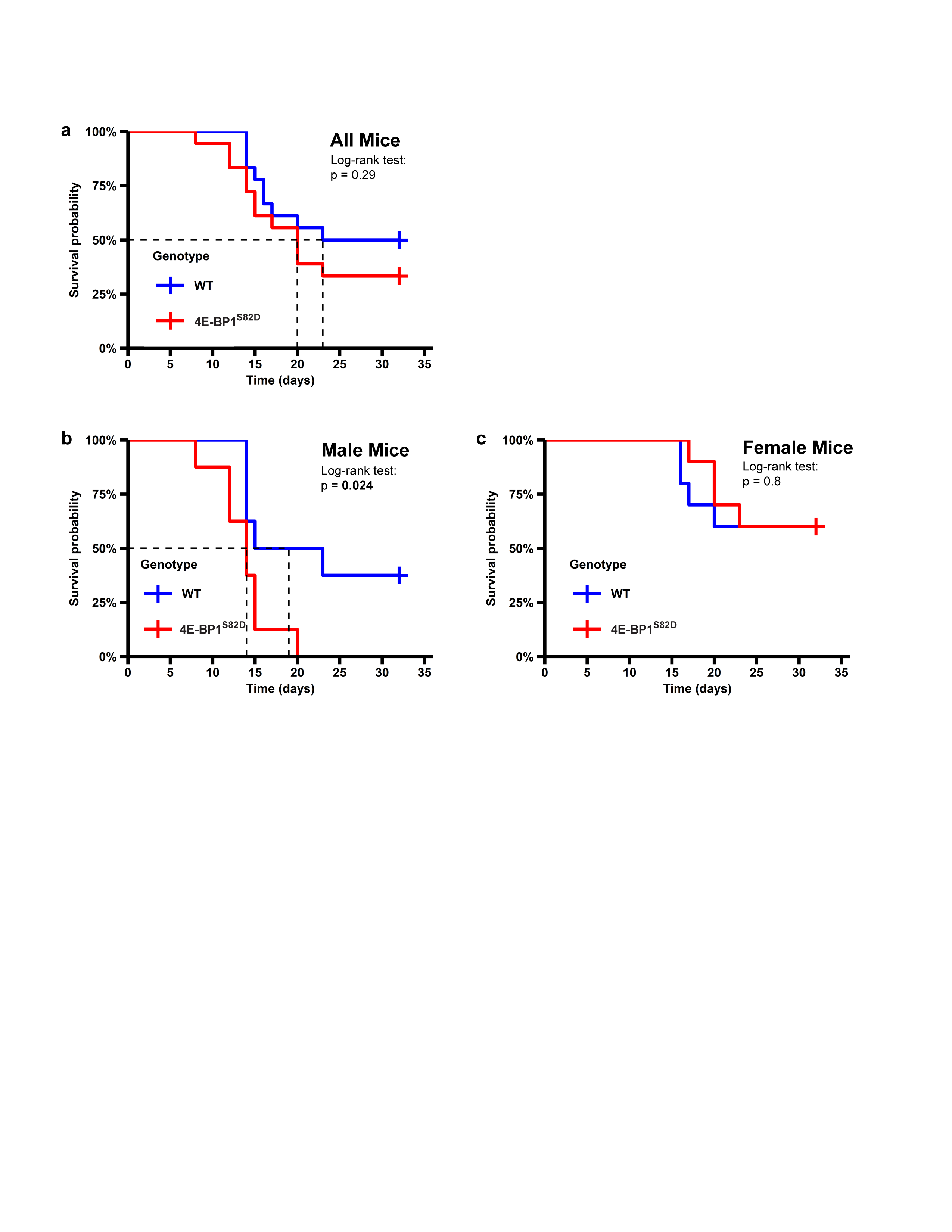

Supplement: S3 Fig — Kaplan-Meier survival curves of a) Male and female 4EBP1S82D mice compared to WT mice (n = 18 per genotype). b) Male 4EBP1S82D mice (n = 8) compared to male WT mice (n = 8). c) Female 4EBP1S82D mice (n = 10) compared to female WT mice (n = 10). Statistical significance determined by log-rank test. (TIF) [file pone.0282914.s003.tif]

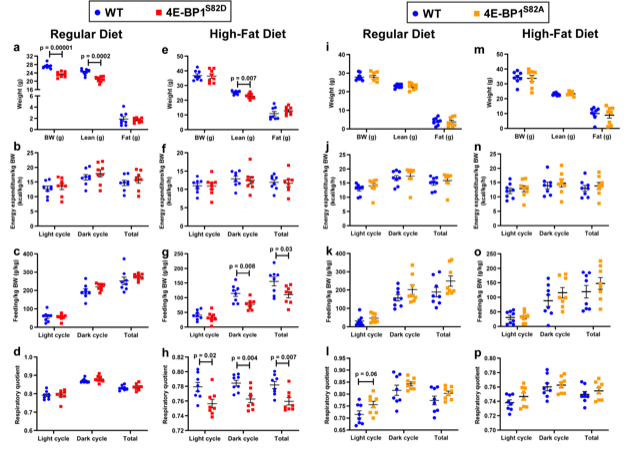

Supplement: S4 Fig — In metabolic cage experiments, male 4E-BP1S82D and WT littermates (n = 8 for each genotype) were fed RCD and were measured for body weight (BW), lean mass, and fat mass (a), energy expenditure per kg BW (b), feeding per kg BW (c), and respiratory quotient per mouse (d). Then, mice previously fed RCD were pre-conditioned with HFD for 6 weeks prior to metabolic analyses and were measured for body weight, lean mass, and fat mass (e), energy expenditure per kg BW (f), feeding per kg BW (g), and respiratory quotient per mouse (h). Metabolic cage experiments were repeated in 4E-BP1S82A and WT littermates fed RCD (i-l) and then HFD (m-p) (n = 8 for each genotype). Mean and SEM are shown, and each plotted point represents one mouse. Two-tailed t-tests were used to compare groups. (TIFF) [file pone.0282914.s004.tiff]

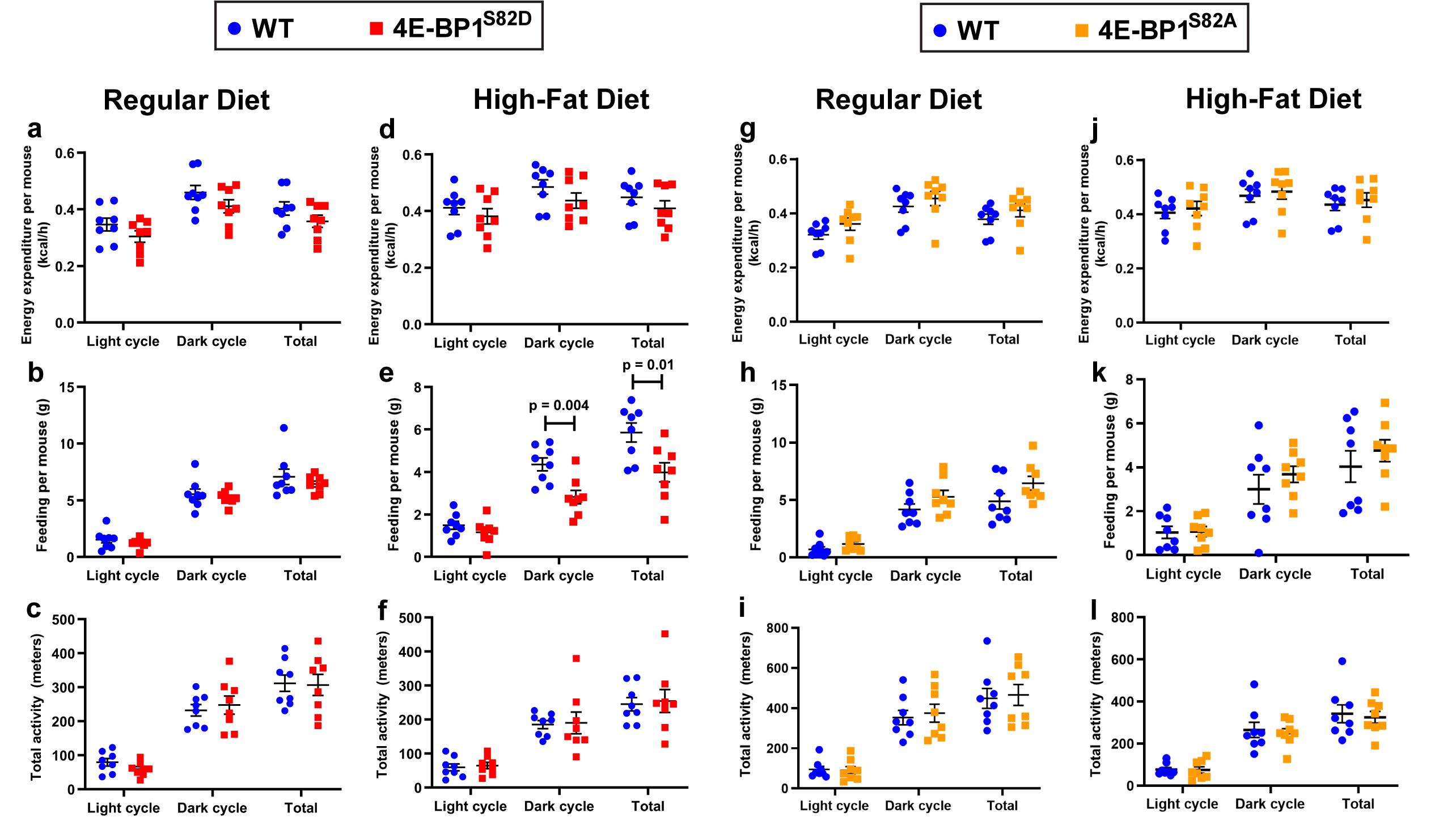

Supplement: S5 Fig — Additional per-mouse metabolic cage data for energy expenditure, feeding, and total activity are shown here to accompany data presented in S4 Fig. Male 4E-BP1S82D and WT littermates (n = 8 for each genotype) were fed RCD and were measured for energy expenditure per mouse (a), feeding per mouse (b), and total activity per mouse (c). After reconditioning on HFD, the mice were measured for energy expenditure per mouse (d), feeding per mouse (e), and total activity per mouse (f). Metabolic cage experiments were repeated in 4E-BP1S82A and WT littermates fed RCD (g-i) and then HFD (j-l) (n = 8 for each genotype). Mean and SEM are shown, and each plotted point represents one mouse. Two-tailed t-tests were used to compare groups. (TIF) [file pone.0282914.s005.tif]

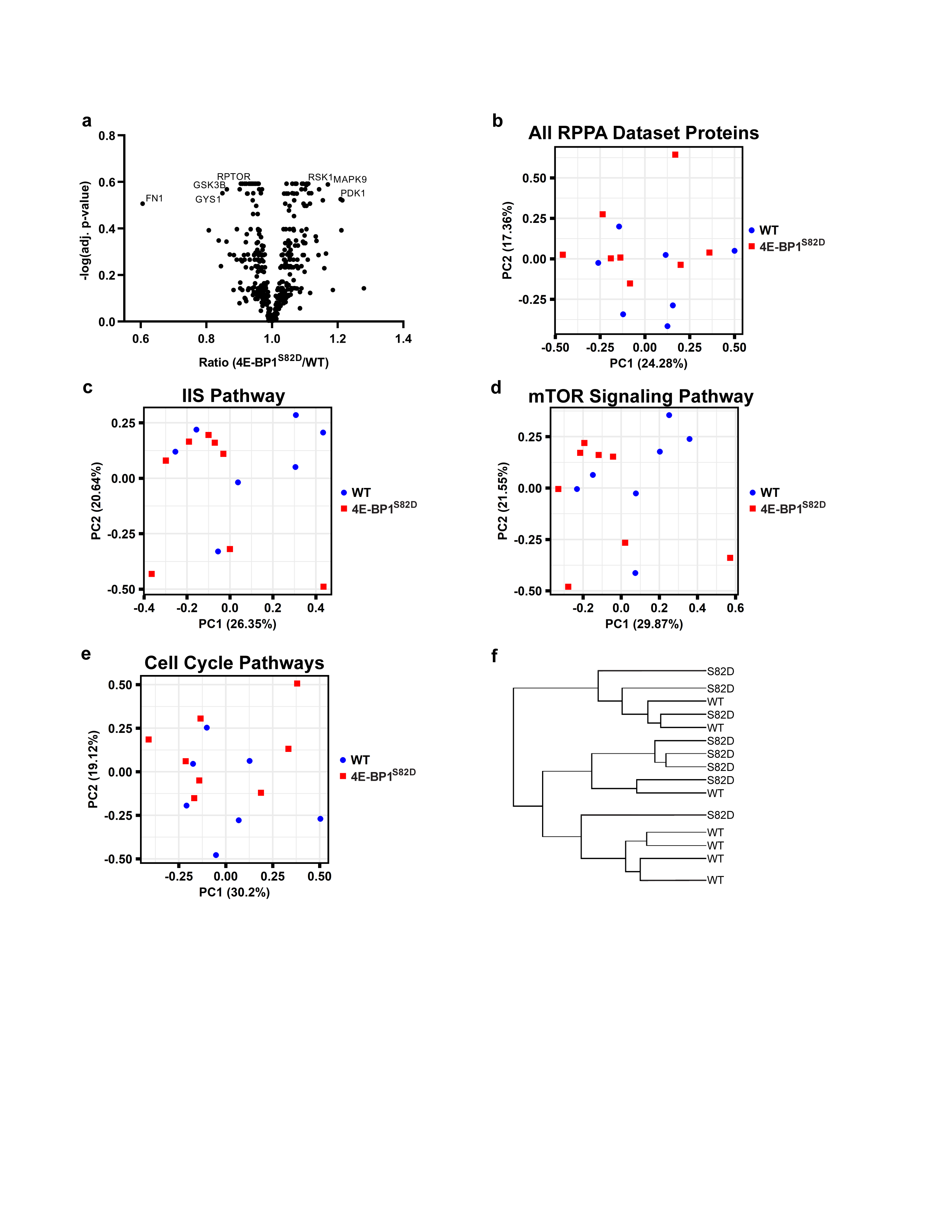

Supplement: S6 Fig — Right gastrocnemius muscle tissue from male mice subject to GTT (4EBP1S82D n = 8, WT n = 7) were used for reverse-phase protein array (RPPA). a) Volcano plot of protein expression by -log(FDR adj. p-value) and expression ratio. b-e) Principal component analysis (PCA) of protein expression in 4EBP1S82D and WT mice from proteins in the full RPPA dataset (b), insulin signaling pathway (IIS, c), mTOR signaling pathway (d), or cell cycle pathways (e). f) Hierarchical clustering of 4EBP1S82D and WT mice based on protein expression from the full RPPA dataset. (TIF) [file pone.0282914.s006.tif]

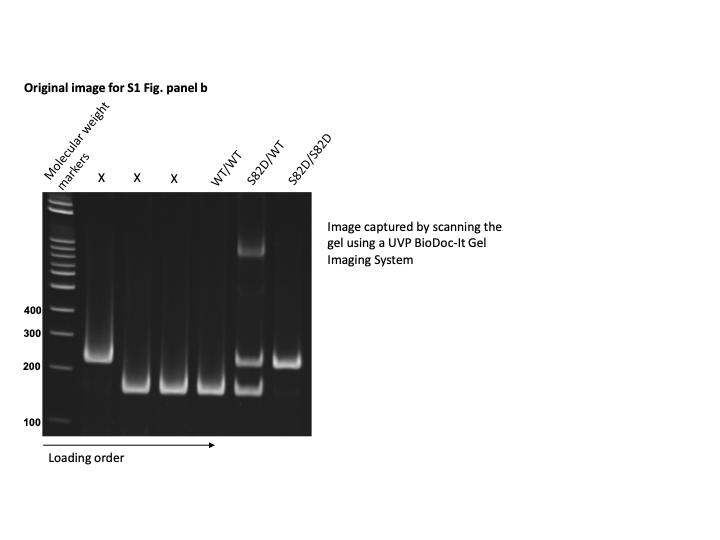

Supplement: S1 Raw images — (TIFF) [file pone.0282914.s009.tiff]
